# Mycoplasma hyopneumoniae Surveillance in Pig Populations: Establishing Sampling Guidelines for Detection in Growing Pigs

**DOI:** 10.1128/JCM.03051-20

**Published:** 2021-04-20

**Authors:** Maria Jose Clavijo, Dapeng Hu, Seth Krantz, Jean Paul Cano, Thairê Pereira Maróstica, Alexandra Henao-Diaz, Ana Paula S. Poeta Silva, Deanne Hemker, Edgar Tapia, Silvia Zimmerman, Eduardo Fano, Dale Polson, Robert Fitzgerald, Alexander Tucker, Rodger Main, Chong Wang, Jeffrey J. Zimmerman, Marisa L. Rotolo

**Affiliations:** a Veterinary Diagnostic and Population Animal Medicine, Iowa State University, Ames, Iowa, USA; b Pig Improvement Company, Hendersonville, Tennessee, USA; c Department of Statistics, Iowa State University, Ames, Iowa, USA; d Tosh Pork, Henry Tennessee, USA; e Pipestone Veterinary Clinic, Pipestone, Minnesota, USA; f Departamento de Clínica e Cirurgia Veterinárias, Universidade Federal de Minas Gerais, Belo Horizonte, Minas Gerais, Brazil; g IDEXX laboratories, Westbrook, Maine, USA; h Boehringer Ingelheim Animal Health USA, Inc., Atlanta, Georgia, USA; i University of Cambridge, Cambridge, United Kingdom; University of Tennessee at Knoxville

**Keywords:** *Mycoplasma hyopneumoniae*, surveillance, oral fluid, probability of detection, tracheal samples, oral fluids, sensitivity, tracheal sample

## Abstract

Antemortem detection of Mycoplasma hyopneumoniae infection in swine production systems has relied on antibody testing, but the availability of tests based on DNA detection and novel diagnostic specimens, e.g., tracheal swabs and oral fluids, has the potential to improve M. hyopneumoniae surveillance. A field study was performed over a 14-week period during which 10 pigs in one pen at the center of a room with 1,250 6-week-old pigs housed in 46 pens were intratracheally inoculated with M. hyopneumoniae. Thereafter, one tracheal sample, four serum samples, and one oral fluid sample were collected from every pen at 2-week intervals. Tracheal and oral fluid samples were tested for M. hyopneumoniae DNA and serum samples for M. hyopneumoniae antibody. Test results were modeled using a hierarchical Bayesian model, based on a latent spatial piecewise exponential survival model, to estimate the probability of detection by within-pen prevalence, number of positive pens in the barn, sample allocation, sample size, and sample type over time. Analysis showed that tracheal samples provided the earliest detection, especially at large sample sizes. While serum samples are more commonly collected and are less expensive to test, high probability of detection estimates were only obtained 30 days postexposure at large sample sizes. In all scenarios, probability of detection estimates for oral fluids within 30 days were significantly lower than those for tracheal and serum samples. Ultimately, the choice of specimen type, sample number, and assay will depend on testing objectives and economics, but the estimates provided here will assist in the design of M. hyopneumoniae surveillance and monitoring programs for different situations.

## Abstract (precis)

Antemortem detection of Mycoplasma hyopneumoniae infection in swine production systems has relied on antibody testing, but the availability of tests based on DNA detection and novel diagnostic specimens, e.g., tracheal swabs and oral fluids, has the potential to improve M. hyopneumoniae surveillance. A field study was performed over a 14-week period during which 10 pigs in one pen at the center of a room with 1,250 6-week-old pigs housed in 46 pens were intratracheally inoculated with M. hyopneumoniae.

## INTRODUCTION

Mycoplasma hyopneumoniae, a linchpin in the porcine respiratory disease complex and the cause of enzootic pneumonia in pigs (1), is one of the most challenging bacterial pathogens in swine production systems. M. hyopneumoniae causes chronic bronchopneumonia, a nonproductive cough, reduced daily weight gain, poor feed conversion, and estimated economic losses of between $0.63 and $10.12 per market pig (2). Within pig populations, transmission of M. hyopneumoniae occurs slowly, primarily through nose-to-nose contact, such that one infected pig can infect 1.16 pigs during a 6-week nursery period (3). This low rate of transmission is one of the most challenging aspects of M. hyopneumoniae disease management and surveillance, especially for early detection in naive populations.

M. hyopneumoniae surveillance may be done using various sampling strategies and testing protocols. Selection of specimen and test depends on the level of disease in the herd, accuracy of the test, and cost. Most commonly, antemortem monitoring of M. hyopneumoniae is done using enzyme-linked immunosorbent assay (ELISA) because of the ease of sample collection, lower cost, and diagnostic performance (4). However, the utility of ELISAs is compromised by the highly variable period between infection and antibody production (∼3 to 8 weeks), the ambiguous relationship between positive results and clinical disease, and the inability to differentiate natural infection from vaccination (3–5).

Alternatively, PCR may be used, but the process of sampling must provide the highest likelihood of the presence of M. hyopneumoniae DNA in the sample. M. hyopneumoniae establishes itself within the lower respiratory tract, mainly the trachea and bronchi, and thus sampling needs to target these sites (6). Several specimens have been evaluated for the detection of M. hyopneumoniae by PCR, including nasal, tonsil, laryngeal, and tracheal samples. A recent study carried out by Sponheim et al. (7) showed a significant increase in diagnostic sensitivity of tracheal samples compared to that of laryngeal swabs. However, collection of tracheal samples requires more time and animal restraint than serum collection. Furthermore, for an individual animal sample, the cost per PCR is roughly six times that of an ELISA.

In contrast to sera and tracheal samples, aggregate specimens, such as oral fluids, require less labor, are less stressful for the pigs, and have demonstrated greater sensitivity versus individual pig samples for the detection of other swine diseases, such as porcine reproductive and respiratory syndrome virus (PRRSV) (8). Previous research suggested that oral fluids could serve as a suitable diagnostic specimen for M. hyopneumoniae, especially during active clinical infection (9).

Surveillance protocols for introduction of negative replacement gilts into naive herds have traditionally relied on the testing of serum samples to validate M. hyopneumoniae-negative herd status. This approach provides M. hyopneumoniae surveillance with minimal labor and expense, but the probability of M. hyopneumoniae detection using this approach is uncertain. Likewise, estimates of the probability of detection for alternative sampling approaches, e.g., tracheal samples and oral fluids, are not available. Therefore, the objective of this study was to estimate the probability of M. hyopneumoniae detection in tracheal samples (DNA), oral fluids (DNA), and sera (antibodies) as a function of M. hyopneumoniae prevalence and sample size.

## MATERIALS AND METHODS

### Animal care and housing.

This study was reviewed and approved by Iowa State University (ISU)’s Institutional Animal Care and Use Committee (IACUC-18-141). The study was conducted on a wean-to-finish site with two connected double-wide barns (2 rooms per barn) housing ∼5,000 pigs. Barns had automatic tunnel ventilation with liquid propane gas brooders for weaned pigs and water misters for heat dispersion. Manure was collected in deep pits, and the site was managed all-in-all-out. The study was carried out in one room (1,250 6-week-old pigs) with 23 pens (∼28 pigs per pen) on either side of a central alleyway. Two pens were kept empty, were half the size of regular pens, and were strictly used as recovery or hospital pens by the production system. The room was stocked with 21-day-old barrows confirmed negative for M. hyopneumoniae, PRRSV, and influenza A virus (IAV), utilizing molecular (VetMax PRRSV NA & EU, VetMax, and Gold SIV detection kits; Thermo Fisher Scientific) and serological assays (IDEXX *M. hyo* Ab test, IDEXX PRRS OF Ab ELISA test, and IDEXX influenza Ab test). During the study, pigs did not receive M. hyopneumoniae vaccine and were not treated with M. hyopneumoniae-susceptible medication. All animal veterinary care, housing, handling, and feeding were under the supervision of production system veterinarians.

### M. hyopneumoniae inoculation and sampling of seeder pigs (inoculated pen).

Seven days prior to the initiation of the experiment, one tracheal, one serum, and one oral fluid sample were collected from each pen from randomly selected pigs to confirm the M. hyopneumoniae-negative status of the room.

To initiate M. hyopneumoniae infection in the room, 10 pigs conveniently selected from a centrally located pen were ear-tagged and intratracheally inoculated with an M. hyopneumoniae lung homogenate (1 × 10^5^ CCU/ml of strain 232, provided by Iowa State University, Ames, IA). Briefly, pigs were immobilized using a snare, and the airway was visualized with a mouth speculum and laryngoscope. A total of 10 ml of lung homogenate was deposited into the trachea via the use of a feeding catheter (10). To confirm M. hyopneumoniae infection, serum and tracheal samples were collected on a weekly basis from every pig in the inoculated pen until each pig was confirmed positive by M. hyopneumoniae ELISA and M. hyopneumoniae PCR, respectively. In addition, one pen-based oral fluid sample was collected weekly from the inoculated pen until the sample was confirmed positive by M. hyopneumoniae PCR.

### Sampling of uninoculated pens.

Beginning at 14 days postinoculation (DPI) and continuing every 14 days over a 14-week period, one tracheal sample, four sera, and one oral fluid sample were collected from each uninoculated pen in the room (*n* = 45). Tracheal samples were collected by restraining the pig with a snare and mouth speculum and introducing a single-use catheter into the trachea, as previously described (11). Serum samples were collected via venipuncture from the jugular vein. Oral fluid samples were collected by suspending a rope from the pen gate such that the end of the rope was level with the pigs’ shoulders. The rope was suspended in the pen for 30 min (12). During each sampling event, samples were collected by first starting at the southeast section of the barn and then moving north until the last pen from that row was sampled. At this point, samplers then moved to the southwest section, moving north until the last pen from that row was sampled. All samplers were blind to test pen results over the course of the study. The first randomly selected pig in each pen was selected for both tracheal and serum samples. This protocol was executed for a total of seven sampling events over a 14-week period.

### Diagnostic testing.

All tracheal samples, sera, and oral fluids were submitted to the Iowa State University Veterinary Diagnostic Laboratory (VDL) for testing by either PCR or ELISA. M. hyopneumoniae DNA from tracheal samples and oral fluids was extracted using the MagMax-96 pathogen RNA/DNA kit (Applied Biosystems, Carlsbad, CA). Extracted DNA was amplified using a PCR TaqMan Fast virus 1-step mastermix (Life Technologies, Carlsbad, CA) with a previously described protocol (13). PCR runs were performed on an ABI Prism 7500 machine (Applied Biosystems, Waltham, MA). A sample with cycle threshold (*C_T_*) values lower than 37 were considered positive; otherwise, samples were considered negative.

M. hyopneumoniae-specific antibodies in all sera were measured utilizing a commercially available enzyme-linked immunosorbent assay (ELISA) test (IDEXX *M. hyo* Ab test). All samples were tested using the same ELISA equipment, namely, plate washer (ELx405; Biotek Instruments, Inc., Winooski, VT), ELISA reader (EMax Plus microplate reader; Molecular Devices, San Jose, CA), and reader software (SoftMax Pro 7.0; Molecular Devices). Following manufacturer’s instructions, antibody concentration was expressed as the ratio of optical densities (OD) from the sample and mean positive (sample to positive [S/P] ratio). Samples with an S/P ratio equal to or greater than 0.3 were considered positive; otherwise, samples were considered negative.

A total of 26 samples from randomly selected PCR-positive tracheal samples were submitted to the ISU VDL for p146 M. hyopneumoniae sequencing to confirm circulation of the challenge strain (M. hyopneumoniae 232) (14).

### Respiratory distress index (SOMO devices).

Five SOMO devices (SoundTalks NV, Precision Livestock Farming, Belgium) were placed equidistant from each other in the alleyway of the room. SOMO 1 was placed at the far north end of the room. A second device, SOMO 2, was placed north of the center of the room. SOMO 3 was placed in the center of the room. SOMO 4 was placed south of the center of the room. SOMO 5 was placed at the south end of the room. These devices continuously recorded sound throughout the trial. Data from these devices provided measurements for temperature, humidity, and a respiratory distress index (RDI). RDI was determined through a proprietary algorithm. The algorithm automatically detected and classified individual coughs. The algorithm differentiated cough from other sounds such as grunts, squeals, and background noise such as fans, gates, and feeders. A threshold was incorporated into the algorithm to signal an RDI alert. In addition, researchers recorded the first observation of coughing during sampling events.

### Statistical analysis.

A hierarchical Bayesian model based on a latent spatial piecewise exponential model (15) was constructed to estimate the spread of M. hyopneumoniae and the diagnostic sensitivities of tracheal and oral fluid PCRs and serum ELISA. Modeling and graphs were performed in R (R program version 3.6.0, package *rjags* 4.8; R core team 2019).

Let i(i=1,2,…,46) denote the *i*^th^ pen in the barn and *n_i_* denote the number of pigs in the *i*^th^ pen. The sampling time points were $0=\tau_{0}<\tau_{1}<\tau_{2}<\ldots <\tau_{K}<\infty$, and the time unit was 2 weeks. Let l(l=1,2,3) denote the diagnostic test methods, 1 for tracheal PCR test, 2 for serum ELISA, and 3 for oral fluid PCR test. Let *u_ijkl_* be the test outcome of pig *j* in pen *i* at the *k*^th^ sampling time using test method *l*. Note that the oral fluid PCR test was a pen-level method, where the term u_i__*__k__3_ represented the oral fluid PCR test result in the pen *i* at the *k*^th^ sampling time. To any of the tests, the outcome was binned to 0 or 1 (negative or positive result). Let *y_ijk_* denote the true infection status of pig *j* in pen *i* at the *k*^th^ sampling time; *y_ijk_* = 1 if it was infected and 0 otherwise. Once a pig was considered infected, it remained infected until the end of the study. If $y_{\mathrm{ij}k_{0}}=1$, then $y_{\mathrm{ij}k_{0}}=1y_{\mathrm{ij}k_{1}}=1$ for all $k_{1}\;\geq \;k_{0}$. Let *t_ij_* denote the unknown time to event of pig *j* in pen *i*, so that $y_{\mathrm{ijk}}=I\left(t_{\mathrm{ij}}\;<\;\tau_{k}\right)$, where *I*(·) is an indicator function that takes a value of 1 when the condition in the parentheses is true and 0 otherwise. The distance between pens was defined to estimate the transmission of M. hyopneumoniae. The barn comprised two rows of pens. Let *d_ii_*_′_ be the distance between pen *i* and *i*′. If pens *i* and *i*′ were adjacent, then *d_ii_*_′_ = 1; otherwise, *d_ii_*_′_ was calculated by Euclidean distance between pens *i* and *i*′. Note that *d_ii_* = 0. Let $\gamma_{1l}$ and $\gamma_{0l}$ denote the diagnostic sensitivity and specificity of the *l*^th^ test and *p_ik_* be the within-pen prevalence of the *i*^th^ pen at the *k*^th^ sampling time. In this model, it was assumed that $\gamma_{0l}=1$ for estimating $\gamma_{1l},l=1,2,3.$

### (i) Model to represent misclassification.

Given the unknown true infection status, the distribution of diagnostic test results was estimated as follows.

For *l* = 1 (tracheal PCR test), let

$$u_{\mathrm{ijk},1}|y_{\mathrm{ijk},1}=1\;∼\;\text{Bernoulli}\left(\gamma_{1,1}\right),\;j=1,\ldots ,n_{i}$$

$$u_{\mathrm{ijk},1}|y_{\mathrm{ijk},1}=0\;∼\;\text{ Bernoulli}\left(0\right),\;j=1,\ldots ,n_{i}$$

To account for delayed detection of M. hyopneumoniae antibodies that are typically detected around 21 to 56 days after exposure (16), a time lag of 21 days (1.5 time units) was implemented; hence, for *l* = 2 (serum ELISA), let

$$u_{\mathrm{ijk},2}|y_{\mathrm{ij}\left(k-1.5\right),2}=1∼\;\text{Bernoulli}\left(\gamma_{1,2}\right),j=1,\ldots ,n_{i}$$

$$u_{\mathrm{ijk},2}|y_{\mathrm{ij}\left(k-1.5\right),2}=0\;∼\;\text{Bernoulli}\left(0\right),\;j=1,\ldots ,n_{i}$$

A lag parameter, *q*, was introduced to account for the delayed detection in oral fluids.

Hence, for *l* = 3 (oral fluid PCR test), let

$$u_{i*k,3}|p_{i,\tau_{k}-q}\;>\;0\;∼\;\text{Bernoulli}\left(\gamma_{1,3}\right)$$

$$u_{i*k,3}|p_{i,\tau_{k}-q}=0\;∼\;\text{Bernoulli}\left(0\right)$$

For each sampling event, four serum ELISA and one tracheal sample PCR result were obtained for each uninoculated pen. The tracheal sample and the first serum sample were collected from the same pig. A new variable was introduced, namely *z_ikm_* ∼ discrete uniform $\left(1,n_{i}\right),m=1,2,3,4,i\neq 35$.

Thus, for inoculated pens (i=35), let

$$u_{35,\mathrm{jk},1}|y_{35,\mathrm{jk},1}=1\;∼\;\text{Bernoulli}\left(\gamma_{1,1}\right),\;j=1,\ldots ,n_{35},\;l=1$$

$$u_{35,\mathrm{jk},2}|y_{35,j\left(k-1.5\right),2}=1\;∼\;\text{Bernoulli}\left(\gamma_{1,2}\right),\;j=1,\ldots ,n_{35},\;l=2$$

For uninoculated pens (1 ≤ i ≤ 46,i≠35), let

$$u_{\mathrm{ijk},1}|y_{\mathrm{ijk},1}=1\;∼\;\text{Bernoulli}\left(\gamma_{1,1}\right),\;j=z_{\mathrm{ikm}},\;m=1,\;l=1$$

$$u_{\mathrm{ijkl},2}|y_{\mathrm{ij}\left(k-1.5\right),2}=1\;∼\;\text{Bernoulli}\left(\gamma_{1,2}\right),\;j=z_{\mathrm{ikm}},\;m=1,\;2,\;3,\;4,\;l=2$$

### (ii) Modified spatial piecewise exponential model.

The Cox proportional hazards model (17) for the time to event is as follows:

$$\lambda \left(t\text{|}x\right)=\lambda_{0}\left(t\right)\exp x^{T}\beta$$

where λ_0_ (*t*) is the baseline hazard function, *x* is a vector of explanatory variables, and *β*is a vector of fixed effect parameters.

In the study, the duration of the study was partitioned by (*K* + 1) sampling time points, $0=\tau_{0}<\tau_{1}<\tau_{2}<\ldots <\tau_{K}<\infty$. Let the $k^{\text{th}}$ interval be $\left(\tau_{k-1},\tau_{k}\right]$, and $\lambda_{\mathrm{ijk}}$ be the hazard of pig *j* in pen *i* being infected in the interval $\left(\tau_{k-1},\tau_{k}\right]$, given that pig was not infected by time τ*_k−1_*. The conditional distribution of $t_{\mathrm{ij}}|y_{\mathrm{ij},k-1}=0$ followed an exponential distribution.

$$t_{\mathrm{ij}}|y_{\mathrm{ij},k-1}=0\;∼\;\exp\left(\lambda_{\mathrm{ijk}}\right),\;t_{\mathrm{ij}}\in \left(\tau_{k-1},\tau_{k}\right]$$

Assume that the pens’ prevalence at time τ*_k−1_*can affect the hazard of pens at time τ*_k_*. To account for the prevalence effect, the prevalence and the spatial distance were introduced as the covariates into the following hazard function:

$$\log\left(\frac{1}{\lambda_{\mathrm{ijk}}}\right)=\beta_{0}\;+\underset{i^{\text{′}}}{\sum }\left(\beta_{1}\;+\;\beta_{2}\exp\left(-d_{ii^{\text{′}}}\right)\right)p_{i^{\text{′}},k-1}$$

where β_0_ is the regression coefficient for the baseline hazard function, β_1_ is a regression coefficient about the effect of pen prevalence but not associated with the spatial distance, and β_2_ is a coefficient considering both the spatial distance and pen prevalence effect. After transformation, the hazard function for the interval was $\left(\tau_{k-1},\tau_{k}\right]$:

(1) $$\lambda_{\mathrm{ijk}}=e^{-\beta_{0}}\exp\left\{-\underset{i^{\prime }}{\sum }\left(\beta_{1}\;+\;\beta_{2}\exp\left(-d_{ii^{\prime }}\right)\right)p_{i^{\prime },k-1}\right\}.$$

Parameter *C* was introduced to control whether a pen was infected or not. Let *s_ijk_* be a threshold parameter where

$$s_{\mathrm{ijk}}=\{\begin{array}{c} t_{\mathrm{ij}},\mathrm{if}y_{\mathrm{ij},k-1}=0\\ 0,\mathrm{if}y_{\mathrm{ij},k-1}=1 \end{array}\}\;$$

It was assumed a pig in a pen can be infected only if the pen was infected. Pen *i* was infected at time point $\tau_{k}$ if ${\bar{s}}_{i*k}\;<\;C$. Then the conditional probability of pig *j* in pen *i* being infected at $\tau_{k}(k\;\geq \;1$) is

$$p\left(y_{\mathrm{ijk}}=1\text{|}p_{\text{*}k-1},\beta ,s_{\mathrm{ijk}}\right)=I\left(y_{\mathrm{ij},k-1}=1\right)+p\left(t_{\mathrm{ij}}\in \left(\tau_{k-1},\tau_{k}\right]\text{|}y_{\mathrm{ij},k-1}=0\right)\text{*}I\left(y_{\mathrm{ij},k-1}=0\right)\text{*}I\left({\bar{s}}_{i.k}\;<\;C\right)$$

The model parameters γ_1,1_, γ_1,2_, γ_1,3_, β_0_, β_1_, β_2_, and *C* were estimated through a hierarchical Bayesian model. The empirical priors of γ_1,1_, γ_1,2_, and γ_1,3_were calculated using a beta-binomial model. The priors for other parameters were noninformative.

## RESULTS

### Confirmation of inoculated pen infection.

All tracheal samples, oral fluids and serum samples collected before inoculation tested negative for M. hyopneumoniae by PCR and ELISA. All seeder pigs were positive by tracheal sample PCR on DPI 7 and by serum ELISA on DPI 42. All seeder pig pen mates were positive by tracheal sample PCR on DPI 21 and by serum ELISA on DPI 70. Pen-level oral fluids were positive by PCR on DPI 56. Two of the seeder pigs died during the course of the study, and their lungs were harvested and submitted to the ISU VDL for analysis. PCR testing, histopathology, and *p146* sequencing (14) confirmed evidence of infection in both sets of lungs by M. hyopneumoniae strain 232.

### Confirmation of uninoculated pen infection.

**FIG 1:**
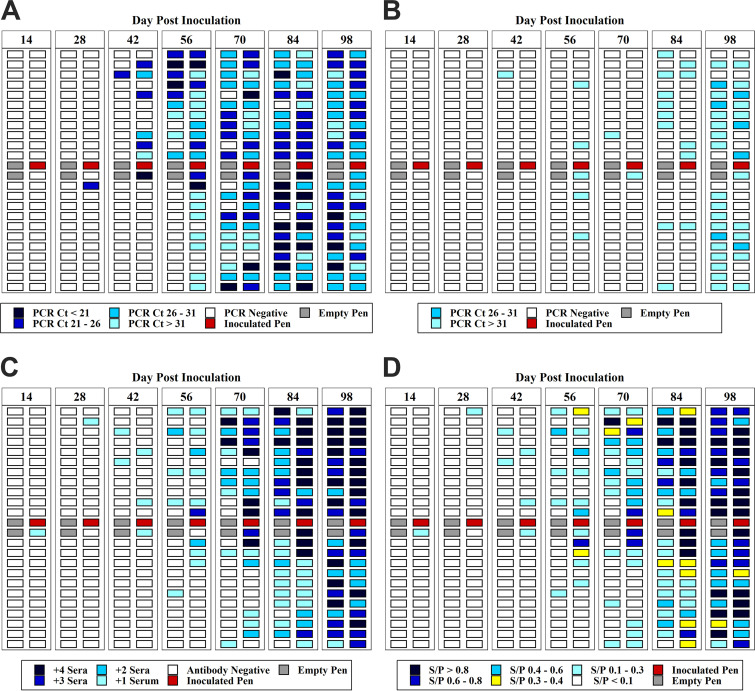
(a) M. hyopneumoniae DNA detection in tracheal samples (range of PCR cycle threshold [*C_T_*] values) over days postinoculation (DPI). (b) M. hyopneumoniae DNA detection in oral fluid samples (range of PCR *C_T_* values) over days postinoculation (DPI). (c) M. hyopneumoniae antibody detection in serum samples (number of positive sera in a pen) over days postinoculation (DPI). Shades of blue represent the number of positive sera per sampling event (0 to 4+). The white boxes represent negative antibody or PCR results. The red boxes represent the inoculated pens. The gray boxes represent the empty recovery pens. (d) Mean range of enzyme-limited immunosorbent assay (ELISA) sample to positive (S/P) ratio values of M. hyopneumoniae antibody by pen of over days postinoculation (DPI).

A total of 315 tracheal samples, 315 oral fluid samples, and 1,258 sera were collected from uninoculated pens during the study. Spatiotemporal detection of M. hyopneumoniae by tracheal samples, oral fluids, and serum samples are shown in Fig. 1a to c. The first detection (1/45) of M. hyopneumoniae by tracheal sample PCR occurred on DPI 28. The initial spread of M. hyopneumoniae began in the pens nearest the inoculated pen and spread north, i.e., the direction of airflow toward the exhaust fans. The infection then progressed to the next row of pens and then to the opposite end of the room. By DPI 56 and 70, 61% (29/45) and 89% (40/45) of the pens, respectively, had at least one positive tracheal sample. *In silico* analysis of 26 randomly selected PCR-positive tracheal samples were confirmed as M. hyopneumoniae strain 232, with a percent identity of >99.8%.

The first detection by ELISA was on DPI 14, with one positive serum result (1/45). By DPI 70, 64% (27/45) of the pens had at least one positive serum result. All pens (45/45) were positive by ELISA on DPI 98 (Fig. 1c). The mean of ELISA S/P values over time is shown in Fig. 1d. Oral fluid PCR results are shown in Fig. 1b. The first positive (1/45) oral fluid sample was detected on DPI 42. By DPI 84 and 98, 40% (18/45) and 78% (35/45) of the pens, respectively, had positive oral fluid results.

### Model parameters and simulations.

The observed field data were used to inform the probability of detection model. The estimated diagnostic sensitivity of tracheal samples was 0.965 (95% credible interval [CI], 0.905 to 0.999). For serum and oral fluid, the diagnostic sensitivity was 0.818 (95% CI, 0.775 to 0.861) and 0.396 (95% CI, 0.285 to 0.507), respectively (Table 1). The parameter estimates, standard errors, and 95% credible intervals are shown in Table 1. The probability of M. hyopneumoniae detection in tracheal samples, oral fluids, and serum samples over time was simulated, at each iteration of which the estimated parameters β_0_, β_1_, β_2_, and *C* were used to simulate the spread of infection and calculate the within-barn prevalence at sampling point $\tau_{k},k\in \{0,1,\ldots ,K\}$. The simulation was initiated with one randomly selected infected pen containing one infected pig. Sampling time (units of 7 days) and sample sizes were specified. The following sampling rules were implemented in the model:

**TABLE 1:** Estimated diagnostic sensitivity of tracheal, serum, and oral fluid samples, and regression model coefficients

Definition	Parameter	Estimate	SE	95% credible interval
Diagnostic sensitivity (Se)				
Tracheal PCR	γ_1,1_	0.965	0.0302	0.905 to 0.999
Serum ELISA	γ_1,2_	0.818	0.0243	0.775 to 0.861
Oral fluid PCR	γ_1,3_	0.396	0.0582	0.285 to 0.507
Hazard baseline	β_0_	1.943	0.1512	1.647 to 2.241
Pen prevalence	β_1_	−0.101	0.0083	−0.117 to −0.085
Spatial and pen prevalence	β_2_	−0.273	0.0406	−0.353 to −0.193
Pen status (0/1)	C	4.743	0.3341	4.118 to 5.374
Lag parameter for oral fluids	q	2.389	0.2451	1.973 to 2.806

A. If the number of samples was less than the number of pens, then each pen had one sample at most.

B. If the number of samples was more than the number of pens, then each pen had at least one sample for each diagnostic test method.

C. The same pig cannot be sampled twice for the same diagnostic test, i.e., for the ELISA; if the number of samples was 50 (exceeding the number of pens), then some pens may have two samples and the two samples are taken from different pigs in that pen.

D. Sampling was memoryless. For example, if a pig was sampled at time τ*_k_* in a simulation iteration, then that pig could also be sampled after τ*_k_* in that simulation iteration.

E. The same pig could be sampled twice for different types of diagnostic test methods at the same time. For example, if a pig was sampled for the serology test method at time τ*_k_*, then this pig could also be sampled for a tracheal PCR test at time τ*_k_*.

**FIG 2:**
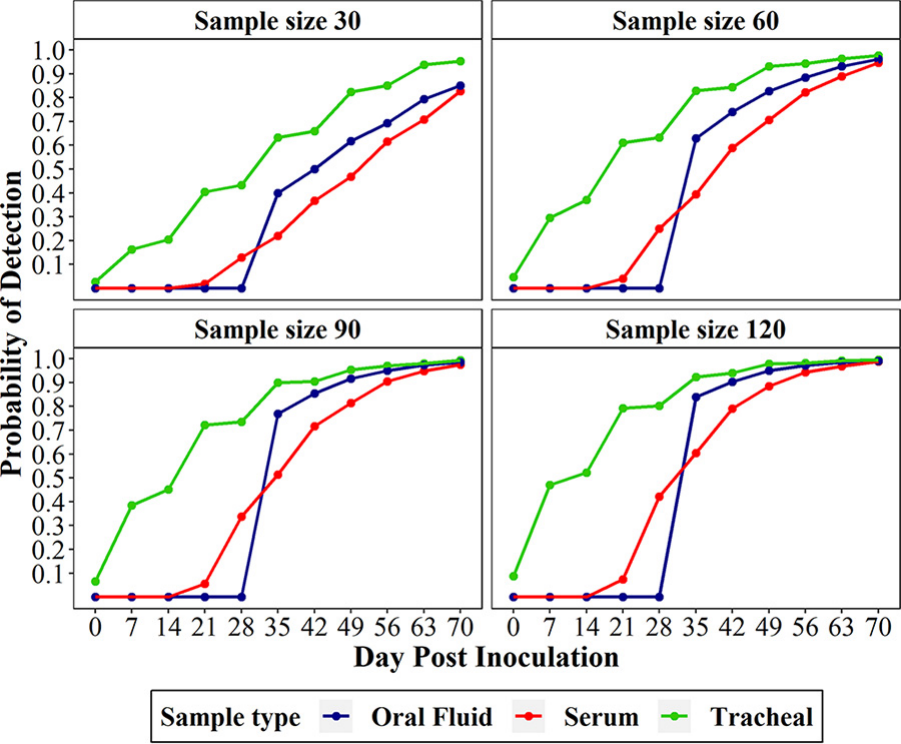
Probability of detection for the first 70 DPI if initial prevalence was one positive pig in a single pen. Probability of detection is given for tracheal samples, serum samples, and oral fluids at samples sizes of 30, 60, and 120.

Given a sample size, samples using the fixed spatial sampling strategy at each sampling time τ*_k_* for each diagnostic test method were selected. Fixed spatial sampling was defined as allocating samples equidistantly across the sampling space. The probability of detection was estimated as the proportion of detection out of 10,000 simulations. This same approach was used to simulate probability of detection over time if five pigs were infected in one pen on DPI 0. These estimates are shown in Table 2 and Fig. 2.

**TABLE 2:** Barn-level probability of detecting at least one positive result using tracheal, serum, and oral fluid samples^*a*^

Sample size (*n*)	No. of initially infected pigs	Sample type^*b*^	No. of days post infection								
			0	7	14	21	28	35	42	49	56
15	1	TS	0.01	0.07	0.14	0.24	0.31	0.44	0.53	0.68	0.75
		SS	0.00	0.00	0.00	0.01	0.07	0.11	0.20	0.27	0.38
		OF	0.00	0.00	0.00	0.00	0.00	0.20	0.28	0.37	0.44
	5	TS	0.06	0.13	0.19	0.30	0.38	0.54	0.63	0.78	0.86
		SS	0.00	0.00	0.00	0.05	0.11	0.17	0.26	0.34	0.48
		OF	0.00	0.00	0.00	0.00	0.00	0.23	0.31	0.42	0.51
30	1	TS	0.03	0.15	0.27	0.42	0.52	0.67	0.76	0.87	0.92
		SS	0.00	0.00	0.00	0.02	0.13	0.22	0.36	0.48	0.61
		OF	0.00	0.00	0.00	0.00	0.00	0.39	0.49	0.61	0.70
	5	TS	0.11	0.24	0.36	0.51	0.62	0.77	0.84	0.93	0.96
		SS	0.00	0.00	0.00	0.10	0.21	0.31	0.46	0.56	0.71
		OF	0.00	0.00	0.00	0.00	0.00	0.42	0.53	0.66	0.75
60	1	TS	0.05	0.28	0.44	0.64	0.75	0.87	0.92	0.96	0.98
		SS	0.00	0.00	0.00	0.04	0.25	0.39	0.58	0.70	0.82
		OF	0.00	0.00	0.00	0.00	0.00	0.63	0.73	0.83	0.89
	5	TS	0.21	0.43	0.57	0.75	0.84	0.93	0.96	0.99	0.99
		SS	0.00	0.00	0.00	0.18	0.39	0.51	0.68	0.79	0.90
		OF	0.00	0.00	0.00	0.00	0.00	0.65	0.77	0.86	0.92
90	1	TS	0.06	0.38	0.57	0.77	0.86	0.94	0.97	0.99	0.99
		SS	0.00	0.00	0.00	0.05	0.34	0.51	0.71	0.81	0.91
		OF	0.00	0.00	0.00	0.00	0.00	0.77	0.86	0.91	0.95
	5	TS	0.31	0.57	0.71	0.86	0.92	0.97	0.99	1.00	1.00
		SS	0.00	0.00	0.00	0.26	0.51	0.66	0.82	0.89	0.95
		OF	0.00	0.00	0.00	0.00	0.00	0.78	0.87	0.94	0.97
120	1	TS	0.09	0.46	0.66	0.84	0.91	0.96	0.98	0.99	1.00
		SS	0.00	0.00	0.00	0.08	0.41	0.60	0.79	0.88	0.95
		OF	0.00	0.00	0.00	0.00	0.00	0.84	0.91	0.95	0.97
	5	TS	0.39	0.67	0.79	0.92	0.96	0.98	0.99	1.00	1.00
		SS	0.00	0.00	0.00	0.34	0.61	0.74	0.88	0.93	0.98
		OF	0.00	0.00	0.00	0.00	0.00	0.86	0.93	0.96	0.98

a As a function of number of pigs initially infected (1 and 5), time, and number of samples collected using a fixed spatial approach.

b TS, tracheal sample; SS, serum sample; OF, oral fluid.

To estimate the point in time probability of detection for tracheal samples and sera, the number of positive pens and the within-pen prevalence was defined. To carry out simulated sampling for the defined number of positive pens and within-pen prevalence, the following rules were applied:

A. If the number of samples was less than the number of pens, then each pen had one sample at most.

B. If the number of samples was more than the number of pens, then each pen had at least one sample for each diagnostic test method.

C. The same pig could not be sampled twice for the same diagnostic test, i.e., for the ELISA; if the number of samples was 50 (exceeding the number of pens), then some pens may have two samples and the two samples are taken from different pigs in that pen.

For each iteration, given the number of positive pens and the within-pen prevalence, the positive pens and the infected pigs were simulated. For example, if there were five positive pens and the within-pen prevalence was 0.2, then five pens were randomly selected to be positive and the infection status of the pigs in those pens followed a Bernoulli distribution (*P* = 0.2). After simulating positive pens and infected pigs, samples were selected based on the sample size. Pens were selected using fixed spatial sampling, while pigs within pens were selected using random sampling. The probability of detection was calculated as the proportion of simulations (out of 10,000) with ≥1 positive pig among the total pigs sampled. Probability of detection estimates are shown in Tables 2 to 4.

**TABLE 3:** Barn-level probability of detecting at least one positive result using tracheal or serum samples^*a*^

Within-pen prevalence (%)	*n*	Probability by sample type of detecting positive result for no. of pens positive (% pens infected)^*b*^:													
		1 (2%)		3 (7%)		5 (11%)		10 (22%)		15 (33%)		25 (56%)		45 (100%)	
		TS	SS	TS	SS	TS	SS	TS	SS	TS	SS	TS	SS	TS	SS
5	5	0.00	0.00	0.02	0.02	0.03	0.02	0.05	0.05	0.08	0.06	0.13	0.11	0.22	0.19
	15	0.02	0.01	0.06	0.05	0.07	0.07	0.13	0.12	0.21	0.18	0.30	0.27	0.52	0.47
	30	0.03	0.03	0.09	0.08	0.17	0.14	0.25	0.20	0.36	0.30	0.57	0.51	0.76	0.72
	60	0.06	0.05	0.16	0.13	0.26	0.24	0.45	0.40	0.62	0.56	0.79	0.74	0.94	0.91
	90	0.08	0.08	0.24	0.21	0.35	0.31	0.61	0.55	0.74	0.68	0.90	0.86	0.99	0.97
	120	0.12	0.10	0.30	0.26	0.44	0.39	0.69	0.64	0.83	0.79	0.95	0.92	1.00	0.99
50	5	0.05	0.04	0.16	0.13	0.23	0.21	0.42	0.37	0.58	0.51	0.79	0.74	0.96	0.93
	15	0.13	0.12	0.40	0.36	0.55	0.49	0.81	0.74	0.92	0.88	0.99	0.98	1.00	1.00
	30	0.27	0.24	0.58	0.52	0.79	0.73	0.96	0.94	0.99	0.98	1.00	1.00	1.00	1.00
	60	0.43	0.39	0.83	0.78	0.95	0.92	1.00	0.99	1.00	1.00	1.00	1.00	1.00	1.00
	90	0.52	0.48	0.90	0.87	0.97	0.95	1.00	1.00	1.00	1.00	1.00	1.00	1.00	1.00
	120	0.56	0.53	0.91	0.89	0.99	0.98	1.00	1.00	1.00	1.00	1.00	1.00	1.00	1.00

a As a function of the within-pen prevalence (5% or 50%), number of pens sampled using a fixed spatial approach (*n*), and number of positive pens in the barn. For serum results, the model assumed that the agent had been in the population at least 21 days.

b TS, tracheal sample; SS, serum sample.

**TABLE 4:** Barn-level probability of detecting at least one positive result using oral fluid samples^*a*^

Ropes	Probability of detecting positive result by no. of positive pens (% pens infected):												
	1 (2%)	2 (4%)	3 (7%)	4 (9%)	5 (11%)	6 (13%)	7 (16%)	8 (18%)	9 (20%)	10 (22%)	15 (33%)	25 (56%)	45 (100%)
1	0.01	0.02	0.03	0.03	0.05	0.05	0.06	0.07	0.08	0.09	0.13	0.22	0.39
2	0.02	0.03	0.05	0.07	0.08	0.10	0.12	0.13	0.15	0.16	0.25	0.38	0.63
3	0.03	0.05	0.08	0.10	0.12	0.15	0.17	0.19	0.22	0.24	0.35	0.52	0.76
4	0.03	0.07	0.10	0.13	0.16	0.19	0.23	0.25	0.28	0.31	0.42	0.62	0.86
5	0.05	0.08	0.13	0.16	0.19	0.24	0.27	0.30	0.32	0.37	0.51	0.71	0.92
6	0.05	0.10	0.15	0.19	0.24	0.29	0.32	0.35	0.39	0.42	0.57	0.78	0.95
7	0.06	0.12	0.17	0.22	0.27	0.32	0.36	0.40	0.44	0.48	0.63	0.82	0.97
8	0.07	0.14	0.20	0.25	0.30	0.35	0.41	0.45	0.49	0.53	0.68	0.86	0.98
9	0.07	0.15	0.20	0.28	0.33	0.39	0.44	0.48	0.53	0.56	0.73	0.89	0.99
10	0.09	0.17	0.24	0.30	0.38	0.42	0.47	0.53	0.56	0.60	0.76	0.92	0.99
15	0.13	0.24	0.34	0.42	0.50	0.58	0.63	0.68	0.72	0.76	0.88	0.98	1.00
20	0.18	0.31	0.44	0.53	0.62	0.68	0.73	0.78	0.82	0.86	0.95	0.99	1.00
25	0.22	0.40	0.52	0.62	0.69	0.77	0.82	0.86	0.90	0.92	0.98	1.00	1.00
30	0.26	0.46	0.60	0.71	0.78	0.83	0.87	0.92	0.93	0.95	0.99	1.00	1.00
35	0.30	0.52	0.66	0.77	0.84	0.89	0.92	0.94	0.96	0.97	1.00	1.00	1.00
40	0.34	0.57	0.72	0.81	0.87	0.93	0.95	0.96	0.98	0.98	1.00	1.00	1.00
45	0.38	0.62	0.77	0.86	0.91	0.94	0.97	0.98	0.99	0.99	1.00	1.00	1.00

a As a function of number of positive pens in the barn and number of pens sampled using a fixed spatial approach.

### Respiratory distress index.

**FIG 3:**
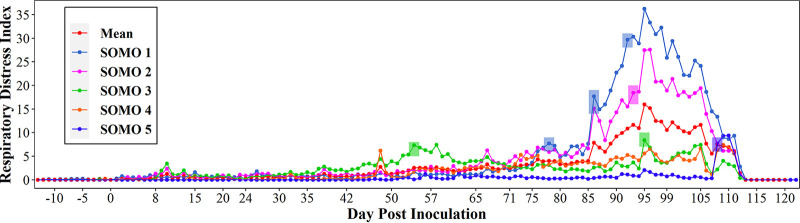
Respiratory distress index (RDI) by SOMO device. Colored rectangles represent RDI alerts.

Figure 3 showed the RDI values recorded during the study for each of the five SOMO devices. The first RDI alert was recorded on DPI 55 by SOMO 3, located in the center of the room nearest to the inoculated pen. On DPI 78, SOMO 1, located at the north end of the barn recorded an RDI alert. Both SOMO 1 and 2, set up on the north end of the room, recorded two RDI alerts on DPI 86. In total, 8 RDI alerts were recorded throughout the study.

## DISCUSSION

Effective surveillance must achieve timely detection of the disease of interest in the target population. A single standardized approach for effective surveillance of all pathogens does not exist; rather, each surveillance program must adopt a pathogen-specific approach that optimizes detection in a cost-effective manner. Due to the elusiveness of M. hyopneumoniae and the limited sensitivity of diagnostic tools, designing an effective M. hyopneumoniae surveillance program is particularly challenging. The foundation of any surveillance program is the estimation of the probability of detection associated with a given combination of sample type, diagnostic test, sample size, sample allocation, and prevalence (8, 18). This is a prerequisite for understanding the effectiveness of a herd surveillance program. Therefore, the objective of this study was to estimate the probability of M. hyopneumoniae detection in tracheal samples, oral fluids, and sera as a function of prevalence, time since initial introduction, and sample size.

Overall, the timing of M. hyopneumoniae detection varied among sample types. Tracheal samples provided the most consistent and earliest detection throughout the study, followed by serum and oral fluids. The M. hyopneumoniae pattern of detection in tracheal samples and sera followed the direction of the airflow, beginning north of the inoculated pen and spreading throughout the south end of the room before spreading to the north end of the room. This observed movement of M. hyopneumoniae may suggest an important role of aerosol transmission within an airspace and its potential implications for early detection. A previous study demonstrated airborne spread of M. hyopneumoniae as a critical risk factor for interfarm transmission (19). However, fomites or pig handlers may have also played a role in the transmission of M. hyopneumoniae in this study.

While the first detection of M. hyopneumoniae was obtained by serum ELISA on DPI 14, M. hyopneumoniae was detected by PCR in tracheal samples in more pens at DPI 42, 56, and 70 compared to detection in serum. These results were expected given the documented lag between infection and production of antibodies (16). Compared to tracheal samples and serum, M. hyopneumoniae detection in oral fluids was delayed, more variable, and less sensitive until DPI 84. Previous research suggested that detection of M. hyopneumoniae in oral fluids was associated with clinical signs, e.g., cough, which may serve to increase the antigen in the oral cavity (9). In this study, cough activity was monitored using SOMO devices. SOMO 3, located in the center of the room, recorded the first RDI alert on DPI 55. The highest average RDI across all SOMO devices was recorded on DPI 95, which coincided with the highest detection of positive pens by oral fluid samples, an observation consistent with the hypothesis proposed by Hernandez-Garcia et al. (9) (Fig. 3).

The field data were modeled to estimate the probability of detection for each sample type, which had not been previously evaluated. M. hyopneumoniae infection was solely determined by tracheal sample and oral fluid PCR and serum ELISA results; the model also provided diagnostic sensitivity estimates for each sample type. In this study, the diagnostic sensitivity estimated for tracheal samples was estimated at 0.965. These results are in accordance with those of a recent study (7). Serum followed, with a sensitivity of 0.818. Recently, diagnostic sensitivity for the IDEXX ELISA was estimated to be 0.56 (20). The noted differences between sensitivities are likely due to the differences in definition of false positives between studies. In this study, a time lag parameter of 21 days was included to account for delayed detection of M. hyopneumoniae antibodies (16). Oral fluids provided the lowest diagnostic sensitivity at 0.396 (Table 1). This number was less than half the diagnostic sensitivity of tracheal and serum samples, supporting the use of these sample types over oral fluid considering current available diagnostic tools.

Two different scenarios (1 versus 5 initially infected animals) were constructed to evaluate the effect of time, sample size, and sample type on the probability of M. hyopneumoniae detection. The number of initially infected animals was chosen to mimic scenarios of very low initial prevalence (0.08% versus 0.4%), and thus the resulting estimates provided in the study were conservative. In scenario 1, 120 tracheal samples collected on DPI 7 provided an ∼50% probability of detection. Furthermore, 120 tracheal samples would provide a probability of detection of ∼96% on DPI 35. In contrast, probability of detection estimates for serum and oral fluid samples could not be generated until DPI 21 and DPI 35, respectively. The earliest detection for serum and oral fluid samples at ∼50% probability of detection occurred with sample sizes of 90 and 60, respectively, on DPI 35. To achieve higher probabilities of detection using serum samples or oral fluids, sample sizes would need to be increased, but time to detection would still be delayed. For example, 120 serum samples would need to be collected at DPI 56 to have a 95% probability of detection. Likewise, 120 oral fluids collected on DPI 49 provided a 95% probability of detection. Interestingly, at DPI 35, 30 oral fluid samples provided almost double the probability of detection (22% versus 39%) as the same number of serum samples (Fig. 2). Reasonably, at an increased initial prevalence of 0.4% (scenario 2), the required sample size and time of detection decreases across sample types; however, tracheal samples remain the optimal sample type. For example, 60 tracheal samples collected on DPI 14 would provide a 57% probability of detection. To achieve 95% probability of detection, 90 tracheal samples on DPI 35 or 120 on DPI 28, respectively, would need to be collected (Table 2).

One of the highest-risk events for a swine production system is the introduction of replacement animals into a herd (21). In most production systems, these animals will undergo a quarantine period (∼30 days) in a separate facility prior to being introduced into the population. During quarantine, it is imperative that diagnostics are performed to determine the health status of the replacement animals. The results of this study support the collection of a high number of tracheal samples for systems that implement a ≤30-day quarantine period (Table 2). In the most conservative scenario, a traditional protocol consisting of 30 serum samples at 28 days post quarantine start would yield a probability of detection of 13% at a cost of $165 (assuming $5.50/ELISA). Given the high risk of introducing potentially positive animals, this traditional approach does not provide a high level of confidence. While doubling the sample size and changing the sample type (i.e., tracheal samples) increases the likelihood of detection by 50%, it also increases the cost 10-fold ($1,800; assuming $30/PCR). For example, collecting ≥60 tracheal samples at 28 days increases the probability of detection to 75%. Despite the significant increase, this sampling approach (i.e., 60 tracheal samples) still does not reach the industry’s traditional expectation of 95% probability of detection (22). Thus, producers should consider extending the quarantine period to 60 days to achieve probabilities of detection closer to 100% using more convenient and economical sample types (i.e., serum and oral fluids).

Selection of sample type and size depends on the level of risk, the impact of the disease, the possibility for extended duration of quarantine, and the cost the operation is willing to pay. For instance, if a production site is experiencing clinical signs suggestive of M. hyopneumoniae or had a recent biosecurity breach, the herd veterinarian will need to decide between collecting and testing a large set of tracheal samples immediately or collecting a smaller set of tracheal, serum, or oral fluid samples at a later time point (Table 2). In the immediate response scenario, the cost of collecting and testing a large set of tracheal samples is high; however, the probability of detection also increases. In the delayed-response scenario, the cost associated with labor and testing is reduced, but this response implies that the risk of delaying diagnosis is minimal.

Probability of detection estimates are necessary for implementation of routine surveillance programs to demonstrate freedom from disease in negative populations. In these cases, veterinarians select a sample size given the desired level of confidence and the cost of sample collection and testing. Testing all animals in a population would provide high confidence in a herd’s negative status, but this approach is impractical. Traditionally, sample sizes have been selected based on estimates provided by Cannon and Roe (22) and Cannon (23), which are based on the approximation of a hypergeometric distribution. The results of this study indicated that 95% probability of detection would require 90 tracheal samples to be collected using a fixed spatial sampling approach from a population of 1,250 in which 50% of the animals across four pens were M. hyopneumoniae positive (Table 3). In contrast, using Cannon’s (23) approximation under the same assumptions would require 65 tracheal samples to be collected using a simple random sampling approach. The underestimation of sample size by Cannon (23) highlights the challenge found in the approximation of the hypergeometric distribution when applied to swine disease surveillance. The differences between the sample sizes are due to the use of a simple hypergeometric model versus the spatial piecewise exponential model. The spatial piecewise exponential model accounts for spatial distance and its relationship with transmission, and is supported by field data. Hypergeometric distribution assumes that the variable of interest, in this case, disease, is randomly distributed with less variation than a spatial model. Furthermore, as demonstrated in previous work, estimates found by Cannon (23) cannot be applied to oral fluid samples, as these are an aggregate sample (18).

Modeling the field data supported previously documented trends related to probability of detection (18, 22). At larger sample size and higher disease prevalence, probability of detection increases for all three sample types. Tracheal samples provided the highest diagnostic sensitivity, earliest detection, and highest probability of detection estimates, but at a significantly higher cost. While the cost associated with increased labor and collection materials incurred in tracheal sampling cannot be reduced, pooling may be an effective approach to decreasing the cost of testing. Future studies should evaluate the effect of pooling schemes on probability of detection. While diagnostic sensitivity of sera was higher than oral fluids, after DPI 35 (scenario 1), oral fluid samples provided a higher probability of detection at every sampling point for the same sample sizes. It is important to point out that increasing the sample size of serum samples at the same time point will achieve similar probability of detection, at a lower testing cost. For example, 60 serum samples, would provide the same 39% probability of detection at a third of the cost of 30 oral fluid samples. While testing pen-based oral fluid samples allows for an increased number of pigs represented in one sample and can be tested for multiple pathogens, future studies should explore potential improvements in M. hyopneumoniae diagnostic performance of this sample type. Probability of detection estimates for all M. hyopneumoniae-specific sample types have been generated by this study and these will be critical in the refinement of current and future M. hyopneumoniae surveillance programs.
